# Happy New Year 2019, and welcome to Conference of Iranian Medical Journal Editors

**DOI:** 10.22038/IJBMS.2018.37088.8837

**Published:** 2019-01

**Authors:** Ali Roohbakhsh, Bizhan Malaekeh-Nikouei, Bibi Sedigheh Fazly Bazzaz

**Affiliations:** 1 Pharmaceutical Research Center, Pharmaceutical Technology Institute, Mashhad University of Medical Sciences, Mashhad, Iran (Assistant Editor); 2 Nanotechnology Research Center, Pharmaceutical Technology Institute, Mashhad University of Medical Sciences, Mashhad, Iran (Assistant Editor); 3 Biotechnology Research Center, Pharmaceutical Technology Institute, Mashhad University of Medical Sciences, Mashhad, Iran (Editor–in–Chief)

The rapid succession of years, and now once again a New Year and a new volume are upon us. In 2018 we celebrated the 20^th^ anniversary of Iranian Journal of Basic Medical Sciences and would like to greet the New Year with our welcome editorial. We leave a great year behind us and are looking forward to a very exciting one ahead. The year 2019 is before us, a year that seemed unimaginable 21 years ago until we were right at its border. The phrase “out with the old and in with the new” is very fitting at this time.

With the continued support of our contributing readership worldwide, we and the editorial team will maintain and improve the status of the journal and its position. Therefore, we look forward to your prized contributions to the journal in 2019 and beyond.

Iranian Journal of Basic Medical Sciences is proud of being an international journal (1) and the leading national multidisciplinary journal in its field. Albeit, being multidisciplinary has consequences particularly for the journal’s Impact Factor and makes competition with highly specialized journals that are very narrow in focus very difficult. 

Up from the **initial 0.324** of just a few years ago (2), we are pleased to report that our impact factor is **now 1.514**, in agreement with the consistent progress of our journal's profile and influence in the scientific community over the past few years. Due to the use of Ithenticate to check integrity and authenticity, the problem of duplicate publication of a few years ago (3) is now under control. With around 1900 submissions in 2018, we are delighted and indebted to our authors and readers for continuing to keep us occupied.

Perhaps due to some upgrades to the IT Department of the University, some e-mails were recently failing to go through. Some Gmail, Hotmail, and Yahoo e-mail addresses were sporadically not receiving notifications, others were being bounced back as undeliverable or sent to junk or spam folders. We were unaware of this problem until notified by some of our members and have been working to fix it. If you use your academic or employer's e-mail address and you are not receiving notifications from IJBMS, please ask your IT department to add MUMS to their safe email list. If you are still not receiving e-mail notifications, especially if you have a Gmail, Hotmail, or Yahoo e-mail address, please contact us to see if the problem can be solved or to provide an alternate e-mail address. We apologize for this inconvenience but look forward to better communications in 2019 and later.

We advise our contributors to revise their acceptable manuscripts as quickly as possible. Our readers will notice the speedy editorial handling process and accordingly the turnaround of submissions of recent issues of Iranian Journal of Basic Medical Sciences over the past 12 months, such that papers are now taking as little as 1 month from receipt to appearance. The main delaying factor is the lengthy duration of revision by some of our authors. In most cases, we have achieved our 1-week target for full-length papers from submission to notification of a decision. The number of submissions during 2018 has increased by more than 30% and we are quite confident that this trend will continue.

Mashhad will be hosting the Conference of Iranian Medical Journal Editors this year. So there is a great opportunity to exchange experiences and share ideas with our colleagues from across the country and international guests. If you are coming to the meeting this year, we look forward to seeing you there.

We are very delighted to take this opportunity to wish our readership and contributors, our editorial team, our independent expert referees, the editorial office, the Vice-chancellor for Research, and the Publications Office of Mashhad University of Medical Sciences a Very Happy New Year, with thanks for all the work they did to make the 21^st^ volume a success.
